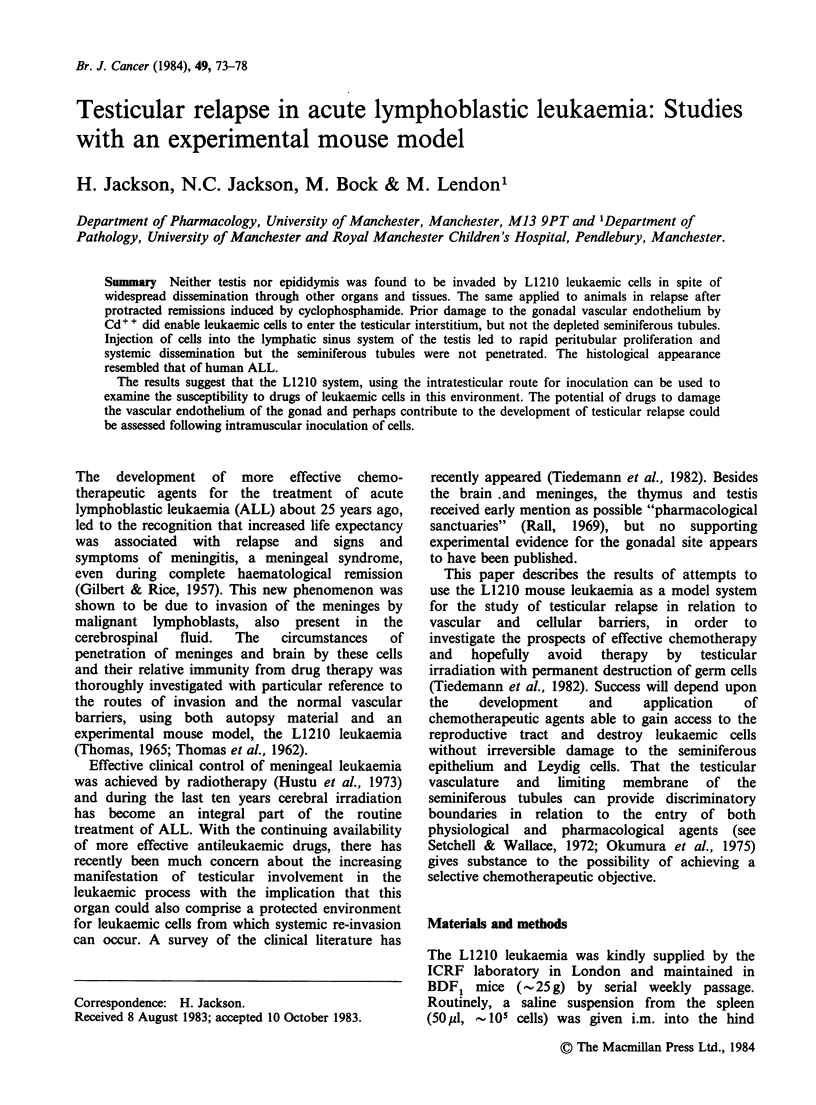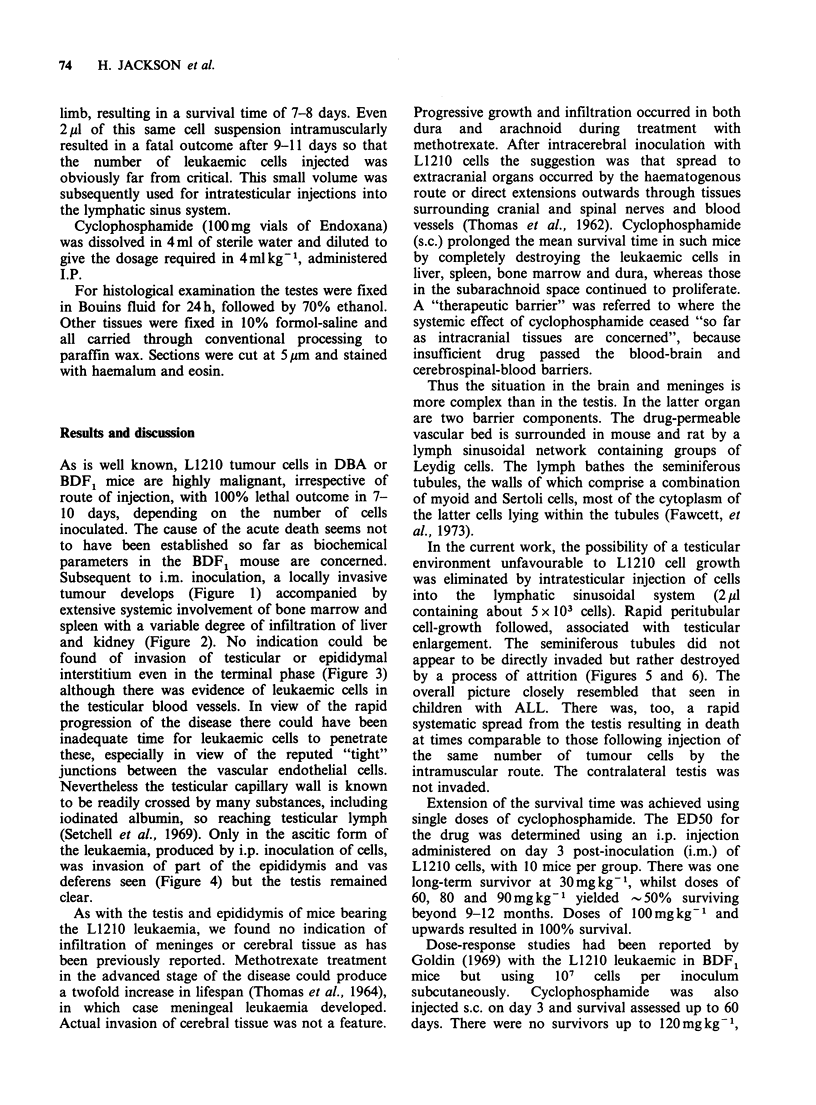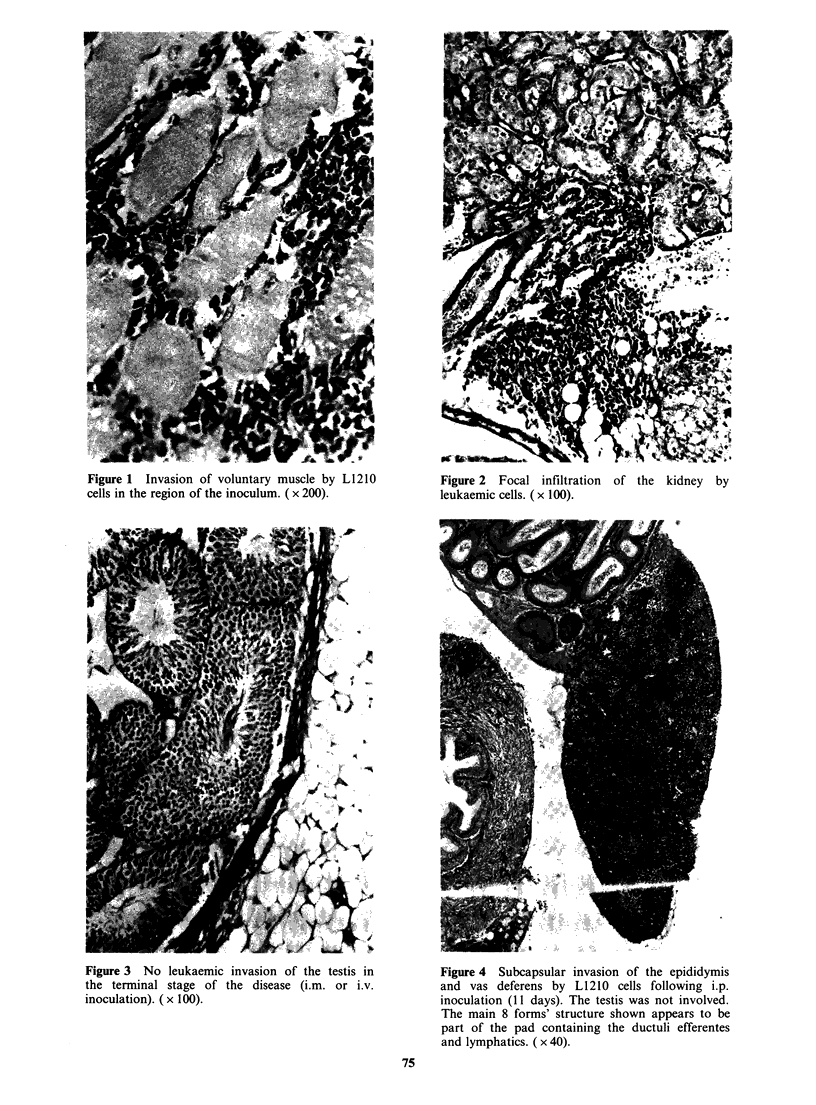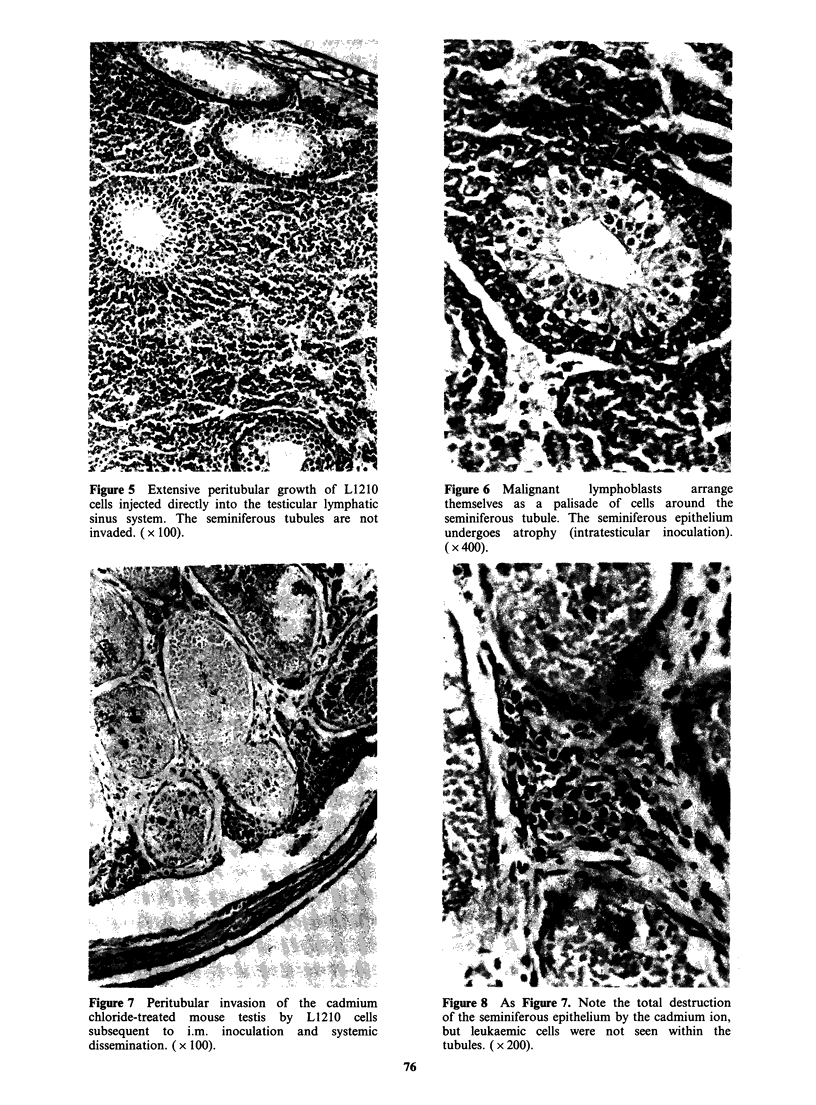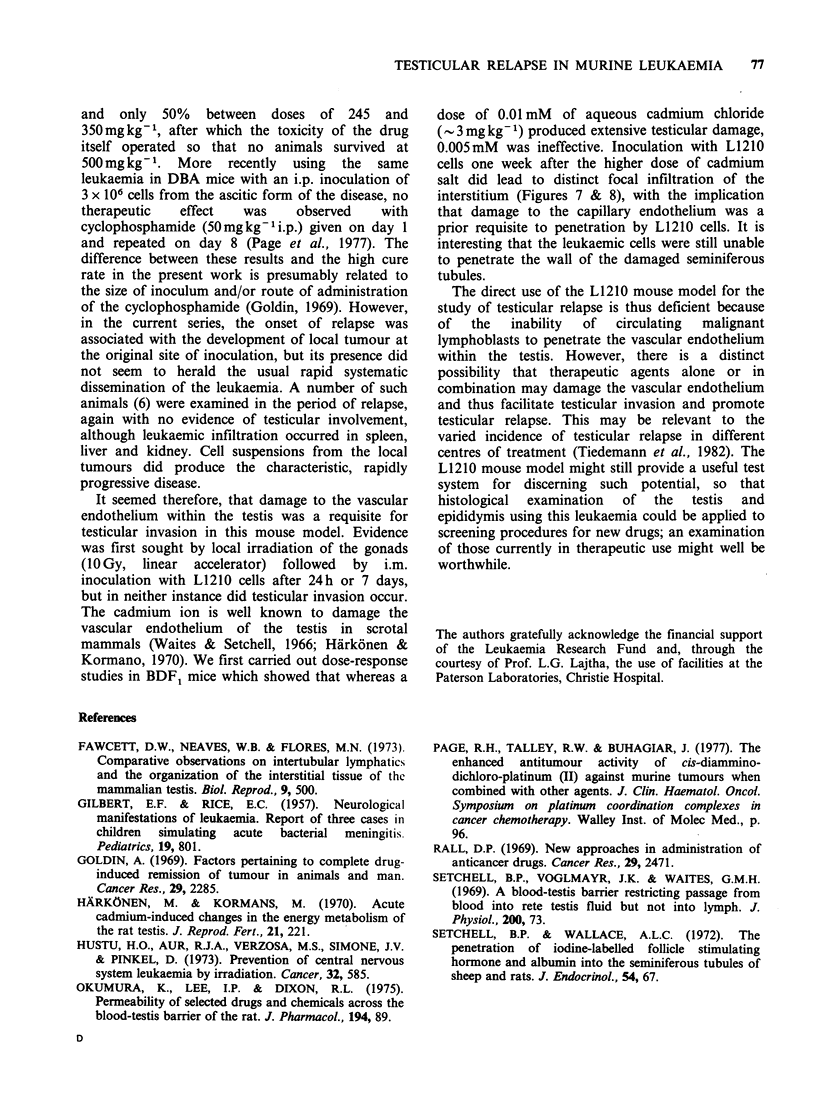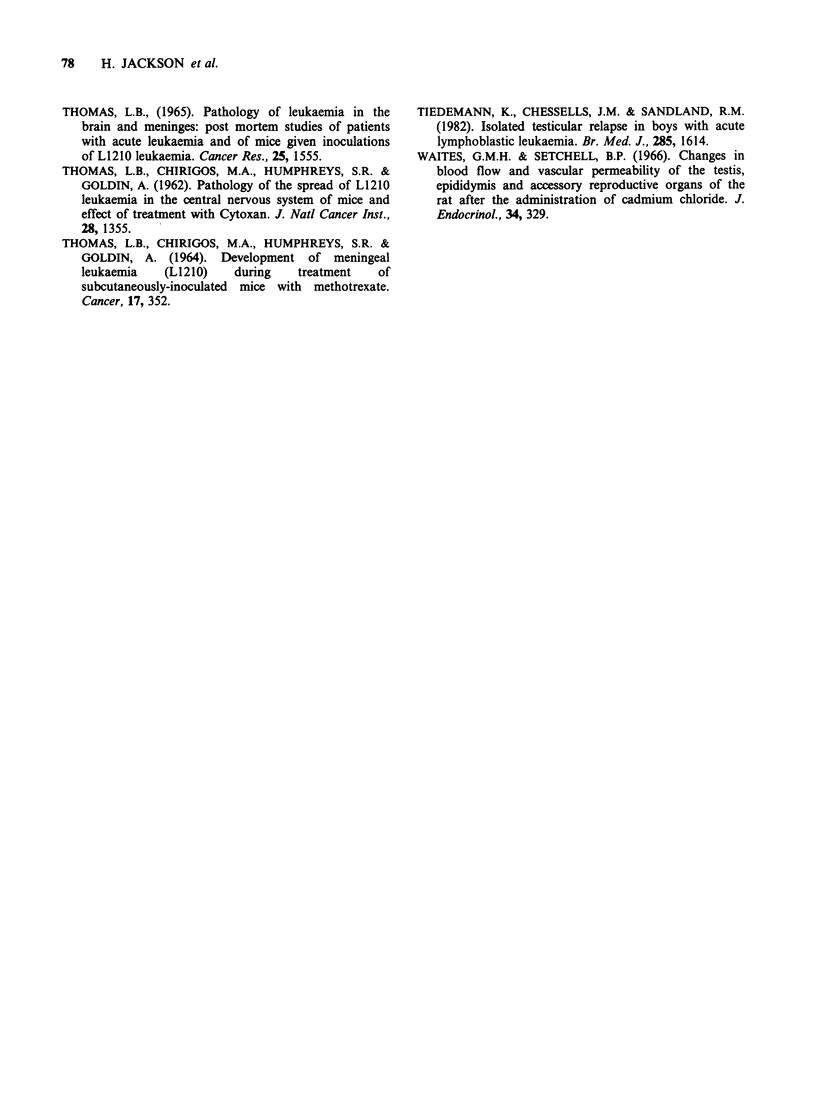# Testicular relapse in acute lymphoblastic leukaemia: studies with an experimental mouse model.

**DOI:** 10.1038/bjc.1984.11

**Published:** 1984-01

**Authors:** H. Jackson, N. C. Jackson, M. Bock, M. Lendon

## Abstract

**Images:**


*Br. J. Cancer* (1984), **49**, 73–78

### Testicular relapse in acute lymphoblastic leukaemia: Studies with an experimental mouse model

H. Jackson, N.C. Jackson, M. Bock & M. Lendon[1]

*Department of Pharmacology, University of Manchester, Manchester, M13 9PT and [1]Department of Pathology, University of Manchester and Royal Manchester Children's Hospital, Pendlebury, Manchester.*

**Summary** Neither testis nor epididymis was found to be invaded by L1210 leukaemic cells in spite of widespread dissemination through other organs and tissues. The same applied to animals in relapse after protracted remissions induced by cyclophosphamide. Prior damage to the gonadal vascular endothelium by $Cd^{++}$ did enable leukaemic cells to enter the testicular interstitium, but not the depleted seminiferous tubules. Injection of cells into the lymphatic sinus system of the testis led to rapid peritubular proliferation and systemic dissemination but the seminiferous tubules were not penetrated. The histological appearance resembled that of human ALL.

The results suggest that the L1210 system, using the intratesticular route for inoculation can be used to examine the susceptibility to drugs of leukaemic cells in this environment. The potential of drugs to damage the vascular endothelium of the gonad and perhaps contribute to the development of testicular relapse could be assessed following intramuscular inoculation of cells.

The development of more effective chemotherapeutic agents for the treatment of acute lymphoblastic leukaemia (ALL) about 25 years ago, led to the recognition that increased life expectancy was associated with relapse and signs and symptoms of meningitis, a meningeal syndrome, even during complete haematological remission (Gilbert & Rice, 1957). This new phenomenon was shown to be due to invasion of the meninges by malignant lymphoblasts, also present in the cerebrospinal fluid. The circumstances of penetration of meninges and brain by these cells and their relative immunity from drug therapy was thoroughly investigated with particular reference to the routes of invasion and the normal vascular barriers, using both autopsy material and an experimental mouse model, the L1210 leukaemia (Thomas, 1965; Thomas *et al.*, 1962).

Effective clinical control of meningeal leukaemia was achieved by radiotherapy (Hustu *et al.*, 1973) and during the last ten years cerebral irradiation has become an integral part of the routine treatment of ALL. With the continuing availability of more effective antileukaemic drugs, there has recently been much concern about the increasing manifestation of testicular involvement in the leukaemic process with the implication that this organ could also comprise a protected environment for leukaemic cells from which systemic re-invasion can occur. A survey of the clinical literature has recently appeared (Tiedemann *et al.*, 1982). Besides the brain and meninges, the thymus and testis received early mention as possible "pharmacological sanctuaries" (Rall, 1969), but no supporting experimental evidence for the gonadal site appears to have been published.

This paper describes the results of attempts to use the L1210 mouse leukaemia as a model system for the study of testicular relapse in relation to vascular and cellular barriers, in order to investigate the prospects of effective chemotherapy and hopefully avoid therapy by testicular irradiation with permanent destruction of germ cells (Tiedemann *et al.*, 1982). Success will depend upon the development and application of chemotherapeutic agents able to gain access to the reproductive tract and destroy leukaemic cells without irreversible damage to the seminiferous epithelium and Leydig cells. That the testicular vasculature and limiting membrane of the seminiferous tubules can provide discriminatory boundaries in relation to the entry of both physiological and pharmacological agents (see Setchell & Wallace, 1972; Okumura *et al.*, 1975) gives substance to the possibility of achieving a selective chemotherapeutic objective.

### Materials and methods

The L1210 leukaemia was kindly supplied by the ICRF laboratory in London and maintained in $BDF_1$ mice ($\sim$25 g) by serial weekly passage. Routinely, a saline suspension from the spleen (50 $\mu$l, $\sim 10^5$ cells) was given i.m. into the hind

Correspondence: H. Jackson.
Received 8 August 1983; accepted 10 October 1983.

© The Macmillan Press Ltd., 1984

limb, resulting in a survival time of 7–8 days. Even 2 $\mu$l of this same cell suspension intramuscularly resulted in a fatal outcome after 9–11 days so that the number of leukaemic cells injected was obviously far from critical. This small volume was subsequently used for intratesticular injections into the lymphatic sinus system.

Cyclophosphamide (100 mg vials of Endoxana) was dissolved in 4 ml of sterile water and diluted to give the dosage required in 4 ml kg$^{-1}$, administered I.P.

For histological examination the testes were fixed in Bouins fluid for 24 h, followed by 70% ethanol. Other tissues were fixed in 10% formol-saline and all carried through conventional processing to paraffin wax. Sections were cut at 5 $\mu$m and stained with haemalum and eosin.

### Results and discussion

As is well known, L1210 tumour cells in DBA or $BDF_1$ mice are highly malignant, irrespective of route of injection, with 100% lethal outcome in 7–10 days, depending on the number of cells inoculated. The cause of the acute death seems not to have been established so far as biochemical parameters in the $BDF_1$ mouse are concerned. Subsequent to i.m. inoculation, a locally invasive tumour develops (Figure 1) accompanied by extensive systemic involvement of bone marrow and spleen with a variable degree of infiltration of liver and kidney (Figure 2). No indication could be found of invasion of testicular or epididymal interstitium even in the terminal phase (Figure 3) although there was evidence of leukaemic cells in the testicular blood vessels. In view of the rapid progression of the disease there could have been inadequate time for leukaemic cells to penetrate these, especially in view of the reputed "tight" junctions between the vascular endothelial cells. Nevertheless the testicular capillary wall is known to be readily crossed by many substances, including iodinated albumin, so reaching testicular lymph (Setchell *et al.*, 1969). Only in the ascitic form of the leukaemia, produced by i.p. inoculation of cells, was invasion of part of the epididymis and vas deferens seen (Figure 4) but the testis remained clear.

As with the testis and epididymis of mice bearing the L1210 leukaemia, we found no indication of infiltration of meninges or cerebral tissue as has been previously reported. Methotrexate treatment in the advanced stage of the disease could produce a twofold increase in lifespan (Thomas *et al.*, 1964), in which case meningeal leukaemia developed. Actual invasion of cerebral tissue was not a feature. Progressive growth and infiltration occurred in both dura and arachnoid during treatment with methotrexate. After intracerebral inoculation with L1210 cells the suggestion was that spread to extracranial organs occurred by the haematogenous route or direct extensions outwards through tissues surrounding cranial and spinal nerves and blood vessels (Thomas *et al.*, 1962). Cyclophosphamide (s.c.) prolonged the mean survival time in such mice by completely destroying the leukaemic cells in liver, spleen, bone marrow and dura, whereas those in the subarachnoid space continued to proliferate. A "therapeutic barrier" was referred to where the systemic effect of cyclophosphamide ceased "so far as intracranial tissues are concerned", because insufficient drug passed the blood-brain and cerebrospinal-blood barriers.

Thus the situation in the brain and meninges is more complex than in the testis. In the latter organ are two barrier components. The drug-permeable vascular bed is surrounded in mouse and rat by a lymph sinusoidal network containing groups of Leydig cells. The lymph bathes the seminiferous tubules, the walls of which comprise a combination of myoid and Sertoli cells, most of the cytoplasm of the latter cells lying within the tubules (Fawcett, *et al.*, 1973).

In the current work, the possibility of a testicular environment unfavourable to L1210 cell growth was eliminated by intratesticular injection of cells into the lymphatic sinusoidal system (2 $\mu$l containing about $5 \times 10^3$ cells). Rapid peritubular cell-growth followed, associated with testicular enlargement. The seminiferous tubules did not appear to be directly invaded but rather destroyed by a process of attrition (Figures 5 and 6). The overall picture closely resembled that seen in children with ALL. There was, too, a rapid systematic spread from the testis resulting in death at times comparable to those following injection of the same number of tumour cells by the intramuscular route. The contralateral testis was not invaded.

Extension of the survival time was achieved using single doses of cyclophosphamide. The ED50 for the drug was determined using an i.p. injection administered on day 3 post-inoculation (i.m.) of L1210 cells, with 10 mice per group. There was one long-term survivor at 30 mg kg$^{-1}$, whilst doses of 60, 80 and 90 mg kg$^{-1}$ yielded $\sim$50% surviving beyond 9–12 months. Doses of 100 mg kg$^{-1}$ and upwards resulted in 100% survival.

Dose-response studies had been reported by Goldin (1969) with the L1210 leukaemic in $BDF_1$ mice but using $10^7$ cells per inoculum subcutaneously. Cyclophosphamide was also injected s.c. on day 3 and survival assessed up to 60 days. There were no survivors up to 120 mg kg$^{-1}$,

**Figure 1** Invasion of voluntary muscle by L1210 cells in the region of the inoculum. ( × 200).

**Figure 2** Focal infiltration of the kidney by leukaemic cells. ( × 100).

**Figure 3** No leukaemic invasion of the testis in the terminal stage of the disease (i.m. or i.v. inoculation). ( × 100).

**Figure 4** Subcapsular invasion of the epididymis and vas deferens by L1210 cells following i.p. inoculation (11 days). The testis was not involved. The main 8 forms' structure shown appears to be part of the pad containing the ductuli efferentes and lymphatics. ( × 40).

**Figure 5** Extensive peritubular growth of L1210 cells injected directly into the testicular lymphatic sinus system. The seminiferous tubules are not invaded. ( × 100).

**Figure 6** Malignant lymphoblasts arrange themselves as a palisade of cells around the seminiferous tubule. The seminiferous epithelium undergoes atrophy (intratesticular inoculation). ( × 400).

**Figure 7** Peritubular invasion of the cadmium chloride-treated mouse testis by L1210 cells subsequent to i.m. inoculation and systemic dissemination. ( × 100).

**Figure 8** As **Figure 7.** Note the total destruction of the seminiferous epithelium by the cadmium ion, but leukaemic cells were not seen within the tubules. ( × 200).

and only 50% between doses of 245 and 350 mg kg$^{-1}$, after which the toxicity of the drug itself operated so that no animals survived at 500 mg kg$^{-1}$. More recently using the same leukaemia in DBA mice with an i.p. inoculation of $3 \times 10^6$ cells from the ascitic form of the disease, no therapeutic effect was observed with cyclophosphamide (50 mg kg$^{-1}$ i.p.) given on day 1 and repeated on day 8 (Page *et al.*, 1977). The difference between these results and the high cure rate in the present work is presumably related to the size of inoculum and/or route of administration of the cyclophosphamide (Goldin, 1969). However, in the current series, the onset of relapse was associated with the development of local tumour at the original site of inoculation, but its presence did not seem to herald the usual rapid systematic dissemination of the leukaemia. A number of such animals (6) were examined in the period of relapse, again with no evidence of testicular involvement, although leukaemic infiltration occurred in spleen, liver and kidney. Cell suspensions from the local tumours did produce the characteristic, rapidly progressive disease.

It seemed therefore, that damage to the vascular endothelium within the testis was a requisite for testicular invasion in this mouse model. Evidence was first sought by local irradiation of the gonads (10 Gy, linear accelerator) followed by i.m. inoculation with L1210 cells after 24 h or 7 days, but in neither instance did testicular invasion occur. The cadmium ion is well known to damage the vascular endothelium of the testis in scrotal mammals (Waites & Setchell, 1966; Härkönen & Kormano, 1970). We first carried out dose-response studies in $BDF_1$ mice which showed that whereas a dose of 0.01 mM of aqueous cadmium chloride (~3 mg kg$^{-1}$) produced extensive testicular damage, 0.005 mM was ineffective. Inoculation with L1210 cells one week after the higher dose of cadmium salt did lead to distinct focal infiltration of the interstitium (Figures 7 & 8), with the implication that damage to the capillary endothelium was a prior requisite to penetration by L1210 cells. It is interesting that the leukaemic cells were still unable to penetrate the wall of the damaged seminiferous tubules.

The direct use of the L1210 mouse model for the study of testicular relapse is thus deficient because of the inability of circulating malignant lymphoblasts to penetrate the vascular endothelium within the testis. However, there is a distinct possibility that therapeutic agents alone or in combination may damage the vascular endothelium and thus facilitate testicular invasion and promote testicular relapse. This may be relevant to the varied incidence of testicular relapse in different centres of treatment (Tiedemann *et al.*, 1982). The L1210 mouse model might still provide a useful test system for discerning such potential, so that histological examination of the testis and epididymis using this leukaemia could be applied to screening procedures for new drugs; an examination of those currently in therapeutic use might well be worthwhile.

The authors gratefully acknowledge the financial support of the Leukaemia Research Fund and, through the courtesy of Prof. L.G. Lajtha, the use of facilities at the Paterson Laboratories, Christie Hospital.

### References

FAWCETT, D.W., NEAVES, W.B. & FLORES, M.N. (1973). Comparative observations on intertubular lymphatics and the organization of the interstitial tissue of the mammalian testis. *Biol. Reprod.*, **9**, 500.

GILBERT, E.F. & RICE, E.C. (1957). Neurological manifestations of leukaemia. Report of three cases in children simulating acute bacterial meningitis. *Pediatrics*, **19**, 801.

GOLDIN, A. (1969). Factors pertaining to complete drug-induced remission of tumour in animals and man. *Cancer Res.*, **29**, 2285.

HÄRKÖNEN, M. & KORMANS, M. (1970). Acute cadmium-induced changes in the energy metabolism of the rat testis. *J. Reprod. Fert.*, **21**, 221.

HUSTU, H.O., AUR, R.J.A., VERZOSA, M.S., SIMONE, J.V. & PINKEL, D. (1973). Prevention of central nervous system leukaemia by irradiation. *Cancer*, **32**, 585.

OKUMURA, K., LEE, I.P. & DIXON, R.L. (1975). Permeability of selected drugs and chemicals across the blood-testis barrier of the rat. *J. Pharmacol.*, **194**, 89.

PAGE, R.H., TALLEY, R.W. & BUHAGIAR, J. (1977). The enhanced antitumour activity of *cis*-diammino-dichloro-platinum (II) against murine tumours when combined with other agents. *J. Clin. Haematol. Oncol. Symposium on platinum coordination complexes in cancer chemotherapy*. Walley Inst. of Molec Med., p. 96.

RALL, D.P. (1969). New approaches in administration of anticancer drugs. *Cancer Res.*, **29**, 2471.

SETCHELL, B.P., VOGLMAYR, J.K. & WAITES, G.M.H. (1969). A blood-testis barrier restricting passage from blood into rete testis fluid but not into lymph. *J. Physiol.*, **200**, 73.

SETCHELL, B.P. & WALLACE, A.L.C. (1972). The penetration of iodine-labelled follicle stimulating hormone and albumin into the seminiferous tubules of sheep and rats. *J. Endocrinol.*, **54**, 67.

THOMAS, L.B., (1965). Pathology of leukaemia in the brain and meninges: post mortem studies of patients with acute leukaemia and of mice given inoculations of L1210 leukaemia. *Cancer Res.*, **25**, 1555.

THOMAS, L.B., CHIRIGOS, M.A., HUMPHREYS, S.R. & GOLDIN, A. (1962). Pathology of the spread of L1210 leukaemia in the central nervous system of mice and effect of treatment with Cytoxan. *J. Natl Cancer Inst.*, **28**, 1355.

THOMAS, L.B., CHIRIGOS, M.A., HUMPHREYS, S.R. & GOLDIN, A. (1964). Development of meningeal leukaemia (L1210) during treatment of subcutaneously-inoculated mice with methotrexate. *Cancer*, **17**, 352.

TIEDEMANN, K., CHESSELLS, J.M. & SANDLAND, R.M. (1982). Isolated testicular relapse in boys with acute lymphoblastic leukaemia. *Br. Med. J.*, **285**, 1614.

WAITES, G.M.H. & SETCHELL, B.P. (1966). Changes in blood flow and vascular permeability of the testis, epididymis and accessory reproductive organs of the rat after the administration of cadmium chloride. *J. Endocrinol.*, **34**, 329.